# Gender Differences of Brain Activity in the Conflicts Based on Implicit Self-Esteem

**DOI:** 10.1371/journal.pone.0037901

**Published:** 2012-05-30

**Authors:** Reiko Miyamoto, Yoshiaki Kikuchi

**Affiliations:** 1 Division of Occupational Therapy, Faculty of Health Sciences, Tokyo Metropolitan University, Tokyo, Japan; 2 Department of Frontier Health Science, Graduate School of Human Health Sciences, Tokyo Metropolitan University, Tokyo, Japan; The University of Melbourne, Australia

## Abstract

There are gender differences in global and domain-specific self-esteem and the incidence of some psychiatric disorders related to self-esteem, suggesting that there are gender differences in the neural basis underlying one's own self-esteem. We investigated gender differences in the brain activity while subjects (14 males and 12 females) performed an implicit self-esteem task, using fMRI. While ventromedial prefrontal cortex (vmPFC) was significantly activated in females, medial and dorsomedial PFC (dmPFC) were activated in males in the incongruent condition (self = negative) compared with the congruent condition (self = positive). Additionally, scores on the explicit self-esteem test were negatively correlated with vmPFC activity in females and positively correlated with dmPFC activity in males. Furthermore, the functional relationships among the regions found by direct gender comparisons were discussed based on the somatic-marker model. These showed that, compared to males, females more firmly store even the incongruent associations as part of their schematic self-knowledge, and such associations automatically activate the neural networks for emotional response and control, in which vmPFC plays a central role. This may explain female cognitive/behavioral traits; females have more tendency to ruminate more often than males, which sometimes results in a prolonged negative affect.

## Introduction

Self-esteem is commonly defined as an individual's sense of self-worth [1]–[Bibr pone.0037901-Bolognini1] [3]. Higher levels of self-esteem have been associated with better coping skills, positive affect, emotional stability, and an increased improvement in quality of life perceptions [4]. On the other hand, low self-esteem has been linked to a number of emotional and behavioral problems [5]–[Bibr pone.0037901-McGlashan1] [7]; low self-esteem strongly correlates with depression in cross- sectional studies [8], and is also an important risk factor for the development of eating disorders [9]. Therefore, it is not without saying that self-esteem is essential for individuals, but also is highly important for their own mental health.

There are gender differences in some aspects of self-esteem. Previous meta-analyses found that, in adolescence, males showed higher global self-esteem than females [10]–[Bibr pone.0037901-Major1] [12]. Moreover, a recent meta-analysis found that there were significant gender differences in domain-specific self-esteem; males scored significantly higher than females on personal self and self-satisfaction self-esteem, and females scored higher than males on behavioral conduct and moral-ethical self-esteem [13]. Personal self-concept is a measure of personality apart from the physical body or relationship to others [14], and self-satisfaction is also a measure of happiness with oneself as a person [15]. Both of the measures overlap with global self-esteem, which favors males. On the other hand, moral-ethical self-concept is a measure of one's perceptions of moral-ethical attributes and satisfactions with one's religion or lack of it [14], and behavioral conduct self-esteem measures an individual's perception of how socially acceptable her behavior is. That is, females develop self-worth, based on feedback from others and relationships with others. Furthermore, a meta-analysis on developmental differences in the self-serving bias showed that females demonstrated a decline in the magnitude of the self-serving bias in early adolescence and the decline became to be significant in adulthood (25–55 ys) compared with males [16]. In addition, females have shown higher susceptibilities than males in psychiatric disorders such as depression [17] [18] and eating disorders [17] which have some relations with self-esteem [5]–[Bibr pone.0037901-McGlashan1] [7], [19]. Thus, there expect to be some gender differences in the neural basis of self-esteem, and it is very important to investigate such neural basis which may explain the higher susceptibilities in females. Although there have been several studies involved in self-esteem using fMRI, they are the studies which investigated the relationships between levels of self-esteem and brain activity [20]–[Bibr pone.0037901-Gyurak1] [Bibr pone.0037901-Onoda1] [Bibr pone.0037901-Eisenberger1] [24], but not the studies on gender differences. Moreover, several fMRI studies investigating gender differences have been reported, but they are the studies involved in empathy [25], emotion recognition [26]–[Bibr pone.0037901-Lee1] [28], threat evaluation [29] and stress response [30], but not self-esteem. Thus, there have been no studies on the gender differences of neural basis underlying self-esteem, to our knowledge.

Explicit measures such as Rosenberg scores have ever been widely used in the studies on self-esteem [31]. However, it may be difficult to investigate the gender-differences of brain activity based on self-esteem evaluated by the explicit measures which are subjective ones. As to the studies on the relationships between brain activity and explicit measures of personality, the results have not been consistent probably because the explicit measures are rather subjective ones [32]–[Bibr pone.0037901-Kim1] [Bibr pone.0037901-Ebmeier1] [Bibr pone.0037901-Johnson1] [36]. On the other hand, consistent results on the brain activity related to implicit measures of attitudes and personality, which are more objective than the explicit ones, have been obtained [37]–[38]. The implicit association test (IAT) has clearly demonstrated the presence of implicit self-esteem without any explicit encouragement to engage in self-evaluation [39]. For most individuals, information about the self is associated with a positive valence. Individuals attribute positive traits or outcomes to internal, stable, and global personal characteristics; whereas, negative traits or outcomes are identified as unrelated to personal characteristics [40]. This attributional bias is known as the self-positivity bias and is one of the most common and robust findings within social psychology [41]. Gender differences have also been documented [42]. An accumulation of research has demonstrated a self-positivity bias in the evaluation of self-associated information [43]–[Bibr pone.0037901-Nuttin1] [Bibr pone.0037901-Nuttin2] [46], and the related forms of implicit self-esteem [39] are remarkable for occurring in the absence of any explicit encouragement to engage in self-evaluative activity. People are unaware of exhibiting implicit self-esteem [44], suggesting that it is a form of self-evaluation that occurs in the absence of conscious self-reflection [39]. In terms of process, implicit self-esteem evaluations are presumably more automatic, meaning that they are relatively more unconscious, unintentional, efficient, and uncontrollable than explicit self-evaluations [47]. The contents of implicit self-evaluations are likely to be more positive than those of explicit self-evaluations. There would be consistent relationships between brain activity and the implicit self-esteem which may be based on such automatic neural processes.

Here, we investigated gender differences in brain activities, measured by event-related functional magnetic resonance imaging (fMRI) of subjects performing the self-esteem IAT, by comparing incongruent situations (e.g., “self is negative”) and congruent situations (e.g., “self is positive”). While the congruent association is based on self-positivity, the incongruent association contradicts it and would cause certain conflicts based on self-esteem. Specifically, we investigated the differential brain activity between the incongruent and congruent situations in each gender group, focusing on the relationships between the brain activity and the explicit and implicit self-esteem, and we performed the direct comparison between males and females in the incongruent vs. congruent situations. The present hypothesis is that there would be some gender differences in the neural substrates underlying self-esteem when responding to the incongruences, and the brain activity in females would be more sensitive and cautious to the incongruences than that in males as suggested by the previous psychological and behavioral findings.

## Methods

### Subjects

Fourteen males (mean age ± SD; 20.6±1.28 yr; range19–23 yr) and 12 females (20.3±1.44 yr; range18–23 yr) participated in the present study. There was no significant difference in age between the two groups (*t* = 0.58, *p* = 0.57, *df* = 24). They were healthy undergraduate or graduate students with no history of significant medical, psychiatric, or neurological disorders. All were right-handed, as determined by the handedness scale [48], and were native Japanese speakers. All gave written informed consent for participation in the study, and ethical approval for the study was obtained from the Tokyo Metropolitan University Research Ethics Committee (No. 06086).

### Rosenberg self-esteem scale

Before fMRI scanning, we assessed the explicit self-esteem in each subject using the Rosenberg self-esteem scale [31], a 10-item self-report measure of global self-esteem. Subjects circled the appropriate number for each statement depending on whether they strongly agreed, agreed, disagreed, or strongly disagreed with it. We summed the ratings assigned to all the items after reverse scoring the positively worded items. Scores ranged from 10 to 40, with higher scores indicating higher self-esteem. A difference between the male and female groups was determined at the 5% level of significance (two sample *t*-test).

### Personality measures

After the scanning, the personality for each subject was indexed by using the NEO Five-Factor Inventory (NEO-FFI), self-report questionnaire that assesses the five personality dimensions of neuroticism, extraversion, openness to experience, agreeableness, and conscientiousness. The 60 items of the NEO-FFI were rated on a five point scale. We examined the two sample t-test between males and females for each personality dimension.

### Implicit Association Test

During fMRI scanning, subjects completed a computer-administered version of the IAT, with stimuli developed (Millisecond Inquisit 2.0; Millisecond Software LLC, Seattle, Washington) for estimating levels of implicit self-esteem. Subjects were asked to sort stimuli representing four concepts (self, other, positive, or negative) into one of two response categories, each of which included two of the four concepts. The usefulness of the IAT in measuring association strength depends on the assumption that when the two concepts that share a response are strongly associated, the sorting task is considerably easier than when the two response-sharing concepts are either weakly associated or bipolar-opposed [49].

**Figure 1:**
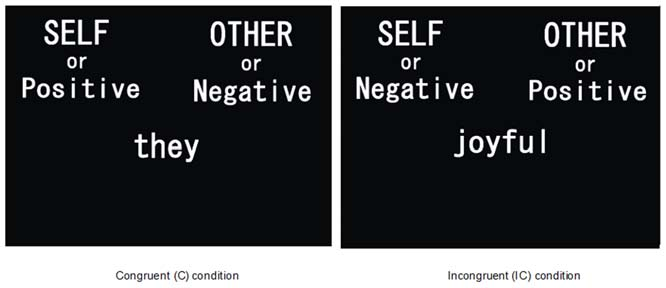
Examples of visual stimuli presented in this experiment. In the real experimental situation, the stimuli were written in Japanese. In each trial the subject was asked to assign the stimulus word to one of the categories given above.

Our experiment was comprised of seven conditions (Table 1). We used 28 words that included 7 positive adjectives (e.g., joyful), 7 negative adjectives (e.g., boring), 7 self-related pronouns (e.g., I) and 7 “other” related pronouns in the third person (e.g., they). We made every attempt to select words with the least difference in emotional context between males and females. The IAT required subjects to categorize “self” pronouns, “other” pronouns, positive words, and negative words as quickly as possible by pressing the appropriate left or right button of a non-magnetic mouse (MRI Compatible Trackball, Resonance Technology, Inc., Northridge, California) with the right index or middle finger, respectively. In one main condition of 28 trials, *self* and *positive* words shared a response key, and *other* and *negative* words shared a key (Fig. 1, Congruent; C, left response). In another main condition of 28 trials, the associations were reversed – *self* with *negative*, and *other* with *positive* (Fig. 1, Incongruent; IC, right response). These trials were presented in random order, and the target word appeared centrally. The duration of each trial was 5 s and the inter-trial interval was 5 s. Before each main condition, we gave subjects practice conditions (conditions 1, 2, 3, 5 and 6; Table 1). Thus, condition 1 was an introduction of the concept condition, conditions 2 and 5 were introductions of the attribute dimension, also in the form of two-category discrimination, and conditions 3 and 6 were for practicing the respective main conditions. Responses during these practice conditions were measured, but were not used for further analysis; subjects did not know that these were practice conditions. Moreover, four conditions (2 and 5 or 4 and 7) were counterbalanced across subjects.

**Table 1:** Example of condition patterns in the Implicit Association Test.

Condition		Concepts for left response	Concepts for right response	Number of trials
1	Practice of concept	self	other	14
2	Practice of attribute	positive	negative	14
3	Practice of Congruent(C)	self or positive	other or negative	28
4	C main	self or positive	other or negative	28
5	Practice of attribute	negative	positive	14
6	Practice of Incongruent (IC)	self or negative	other or positive	28
7	IC main	self or negative	other or positive	28

A pair of concepts was introduced in the first task by asking subjects to respond with the left key (pushed with index finger) to words representing ‘self’ and with the right key (pushed with middle finger) to words representing ‘other’. The fifth task was the reverse of the second. The IAT effect measure was constructed by comparing performance in the 4th and 7th conditions. A more rapid response in the C task than in the IC task indicated that the self-positive and other-negative associations were stronger than the self-negative and other-positive associations.

The total duration of fMRI scanning was 848 s. Instruction slides were shown for 10 s before each of the conditions to instruct the subject how to categorize the word. The IAT tasks were projected onto a screen by a liquid crystal display projector and were seen through a mirror placed above the subject's eyes. The tasks were shown at a horizontal visual angle of 27.8° and vertical visual angle of 18.0°. The subjects were told “This investigation is a test to measure your speed and accuracy in word classification” so that they should not think that their self-esteem was being evaluated.

All response times were recorded using a personal computer. The mean latency for the C condition was subtracted from that for the IC condition for each subject (IAT scores). We analyzed the differences between IC and C conditions at the 5% level of significance for males and females (paired *t*-test). These IAT scores reflect the differential ease with which subjects associate the *“self”* and *“other”* recognition with positive concepts compared with negative concepts. The IAT scores were compared between males and females using the Welch's t-test.

### fMRI data acquisition

Event-related fMRI was conducted using a 1.5-Tesla whole-body superconducting scanner system (Signa Horizon LX, General Electric, Milwaukee, Wisconsin) equipped with a quadrature detection head coil of the birdcage type and an actively shielded gradient coil. The functional scans were performed using a gradient-recalled echo, echo-planar imaging MR sequence and T2*-weighted images were acquired. The BOLD-sensitive single-shot EPI sequence parameters were as follows: TR = 4000 ms, TE = 90.5 ms, flip angle = 80°, matrix size = 128×128 pixels, FOV = 240×240 mm^2^, slice numbers = 20, and slice thickness = 6.0 mm. In addition, T1-weighted anatomic MRI using a magnetization-prepared, fast-spoiled gradient-recalled echo (Fast SPGR) 2D sequence was performed (axial plane, TR = 26.0 ms, TE = 2.4 ms, flip angle = 30°, matrix size = 256×256 pixels, slice thickness = 2.3 mm, FOV = 240×240 mm^2^).

### Statistical analysis

All functional imaging data were preprocessed and analyzed using SPM2 (Welcome Department of Imaging Neuroscience, London, UK; http://www.fil.ion.ucl.ad.uk/spm) implemented in MATLAB 7.0.1 (The Mathworks Inc., Natick, MA, USA.). The functional images were realigned to correct for head movements and were coregistered with the Montreal Neurological Institute (MNI) template. All functional images were spatially filtered by a low-pass Gaussian filter (FWHM = 10 mm) and smoothed to conform to the Gaussian assumptions of SPM and to improve the signal-to-noise ratio. The data were temporally convolved with the hemodynamic response function and high-pass filtered with a cutoff period of 128 s.

Fixed effects analysis was performed on each subject's data, and regressors were included as factors that accounted for the IC condition (condition 7; Table 1) and C condition (condition 4; Table 1). The results of these analyses were then submitted to a second-level, random effect analysis, with subjects in each gender group as the random variable. Statistical activation maps were constructed based on differences between the IC and C conditions (hereafter; IC vs. C condition) for each gender group using a *t*-statistic. These maps were thresholded at *t*>4.12 (*p*<0.001, uncorrected). Moreover, a region was considered significant only if its volume was more than 63 mm^3^. This double threshold corresponds to a 5% multiple comparisons adjusted probability of falsely identifying one or more activated voxel clusters on the basis of Monte Carlo simulations (Alphasim/AFNI (http://afni.nimh.nih.gov/afni/doc/manual/AlphaSim)). Moreover, brain activity differences between gender groups were investigated based on a two-sample *t*-test with a volume threshold of 63 mm^3^ (*p*<0.001, uncorrected).

### Correlation analyses

To assess the relationship between explicit self-esteem and implicit self-esteem, we analyzed the correlation between the IAT scores and the Rosenberg scores for each group at *p*<0.05 (*t*-test for Pearson product-moment correlation coefficient). Furthermore, correlational analyses for the male and female groups were performed, at *p*<0.05, between the IAT and Rosenberg scores and the averaged parameter estimates in the spherical region of interest (ROI; radius, 5 mm), the center of which was the peak voxel in each cluster showing significant activity in the IC condition compared with the C condition at *t*>4.12 (*p*<0.001, uncorrected with a volume threshold of 63 mm^3^).

## Results

### Rosenberg self-esteem scale scores and personality measures

**Figure 2:**
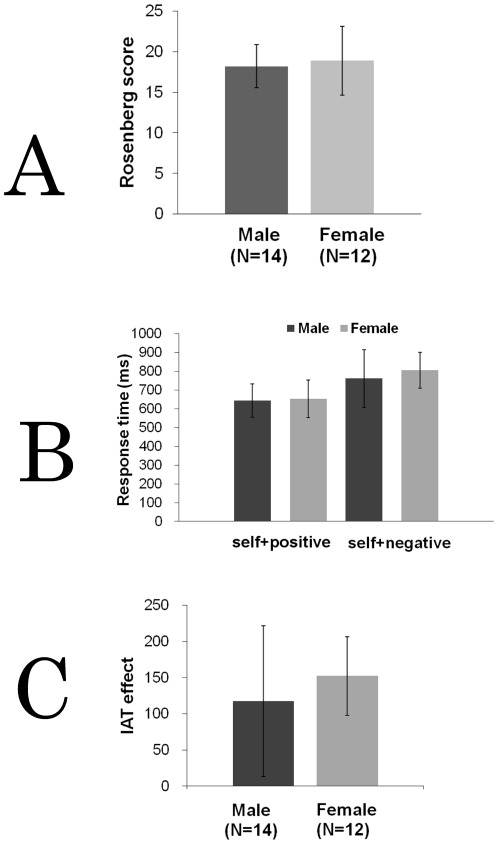
Comparisons of Rosenberg scores, response times in IAT, and IAT effects in males and females. (A) Rosenberg self-esteem scores in males and females. There was no significant gender difference. (B) Response times for each main condition in IAT. (C) The mean IAT scores in males and females. There was no gender difference in the IAT scores. Error bars indicate standard error.

The Rosenberg scores ranged from 14 to 22 in males (mean±SD = 18.2±2.67) and from 14 to 28 in females (18.9±4.25), with no significant gender difference (*t* = −0.51, *p* = 0.61, *df* = 24, Fig. 2A). NEO-FFI *t*-scores ranged from 25 to 55 for neuroticism (mean ± SD =  41.0±8.11), from 41 to 61 for extraversion (51.8±6.76), from 40 to 60 for openness (47.9±7.97), from 31 to 63 for agreeableness (50.5±10.0), and from 28 to 69 for conscientiousness (53.9±11.3) in males. On the other hand, in females, scores ranged from 25 to 65 for neuroticism (45.5±11.0), from 37 to 71 for extraversion (53.9±9.27), from 28 to 71 for openness (51.6±12.2), from 43 to 72 for agreeableness (54.3±9.45), and from 36 to 75 for conscientiousness (55.1±10.6).

There were no significant differences between males and females in either of the scores; neuroticism (t = −1.20, P = 0.24, df = 24), extroversion (t = −0.68, P = 0.51, df = 24), openness (t = −0.92, P = 0.37, df = 24), agreeableness (t = −1.00, P = 0.33, df = 24), and conscientiousness (t = −0.29, P = 0.78, df = 24).

### IAT scores and their correlations with Rosenberg scores

Results from the IAT indicated that in each group the subjects responded significantly faster in the C condition (males: mean±SD = 643±89.0 ms, *t* = 4.25, *p* = 0.02<0.05, *df* = 13; females: 652±100 ms, *t* = 9.70, *p* = 0.0009<0.001, *df* = 11) than in the IC condition (males: 761±154 ms; females: 805±95.4 ms), demonstrating that on average subjects more strongly associated *self* with positive than with negative adjectives. In short, the subjects exhibited an IAT effect (Fig. 2B). In addition, the IAT scores (IC-C) ranged from 9.46 to 433 ms (mean±SD = 118±104 ms) in the male group and from 68.4 to 256 ms (152±54.4 ms) in the female group. There was no gender difference in IAT scores (*t* = −1.09, *p* = 0.228, df = 20, Fig. 2C). We did not find significant correlations between the IAT scores and the Rosenberg scores in either group (males, *t* = −0.65, p = 0.53; df = 12; females, *t* = −0.03, p = 0.98, df = 10).

### Brain activity in each gender group

Brain regions that were significantly activated (significant differential BOLD response) in males in the IC vs. C condition were as follows: the left middle frontal gyrus (MFG), medial prefrontal cortex (mPFC), posterior cingulate cortex, posterior superior temporal gyrus, parahippocampal gyrus, right dorsomedial prefrontal cortex (dmPFC), and cerebellum. On the other hand, the regions that showed a significant differential BOLD response to IC relative to C conditions in females included the left ventromedial prefrontal cortex (vmPFC), inferior parietal lobule (IPL), precuneus, middle occipital gyrus, fusiform gyrus, right superior frontal gyrus, anterior cingulate cortex (ACC), superior parietal lobule, and superior occipital gyrus (Table 2).

**Table 2:** Brain regions with significant activation during IC vs. C.

Anatomical region	Males					Females				
	Coordinates (mm)			t	Volume (mm^3^)	Coordinates (mm)			t	Volume (mm^3^)
	x	y	z			x	y	z		
R superior frontal gyrus						20	−10	74	5.53	240
L middle frontal gyrus	−30	−4	56	6.55	2976					
R dorsomedial prefrontal cortex	20	36	28	4.23	64					
L medial prefrontal cortex	−32	36	14	4.42	224					
L ventromedial prefrontal cortex						−24	38	4	5.85	496
R anterior cingulate cortex						4	8	24	4.73	64
L posterior cingulate cortex	−8	−50	34	4.58	336					
R superior parietal lobule						26	−76	46	5.1	672
L inferior parietal lobule						−50	−50	36	4.3	96
L posterior superior temporal gyrus	−50	−54	10	5.96	1088					
L parahippocampal gyrus	−16	−34	−20	5.42	288					
L precuneus						−26	−78	38	4.18	80
R superior occipital gyrus						30	−68	30	4.75	288
L middle occipital gyrus						−26	−86	10	4.84	176
L fusiform gyrus						−42	−64	−16	4.41	64
R cerebellum	6	−46	−24	6.63	864					

P<0.001, uncorrected, Volume>63 mm^3^, R: right hemisphere, L: left hemisphere, MNI: Montreal Neurological Institute, x, y, z: anatomical coordinates based on the Montreal Neurological Institute brain template.

### Correlations of neural activity with the IAT and Rosenberg scores

**Figure 3:**
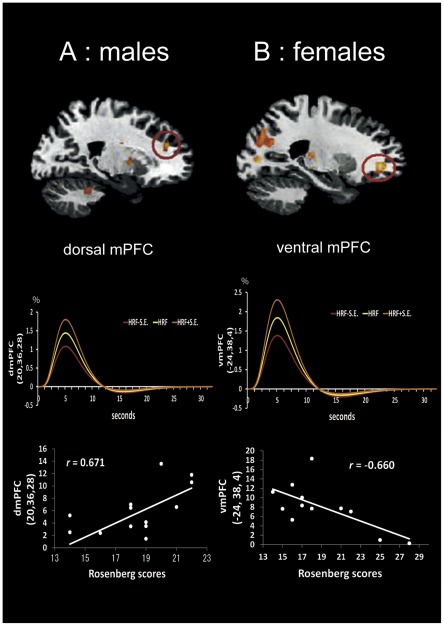
Relationship between Rosenberg scores and the differential activities of mPFC regions for IC vs C condition. (A) *Upper*; the red circle shows the differential activity in the right dmPFC for the IC vs. C conditions in males (x = 20). *Middle*; the hemodynamic response function (HRF) (yellow) averaged across subjects with standard errors (S.E.) recorded from dmPFC in males (y-axis: %, x-axis: seconds). *Lower*; scatter plot depicting the positive correlation between the difference in parameter estimates between the IC and C conditions in the right dmPFC and the Rosenberg self-esteem scores in males (y-axis: arbitrary unit). (B) *Upper*; the red circle shows the differential activity in the left vmPFC for IC vs. C conditions in females (x = −24). *Middle*; the HRF averaged across subjects with S.E. recorded from vmPFC in females (y-axis: %, x-axis: seconds). *Lower*; scatter plot showing the negative correlation between the difference in parameter estimates between the IC and C conditions in the left vmPFC and the self-esteem scores in females (y-axis: arbitarary unit). (p<0.005 uncorrected, for illustration).

In males, activity in the right dmPFC in the IC vs. C condition positively correlated with the Rosenberg score (*r* = 0.67, p<0.01, df = 12, Fig. 3A). On the other hand, the correlation analysis revealed that the Rosenberg score was negatively correlated with the BOLD response in the left vmPFC in females (*r* = −0.66, p<0.05, df = 10, Fig. 3B). There were no activities in other brain regions that showed significant correlations with the Rosenberg scores in either gender group. Furthermore, there were no other brain region activities that significantly correlated with the IAT scores in either gender group.

### Brain activity differences between the male and female groups

**Figure 4:**
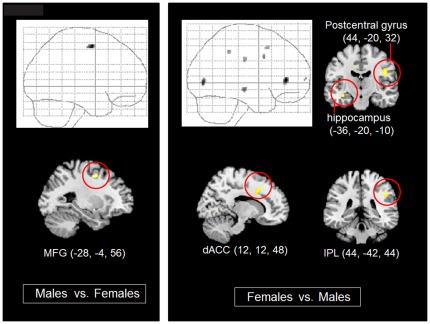
Gender differences in brain activities in the IC vs. C conditions. *Left*; glass brain (*upper*) and Montreal Neurological Institute (MNI) brain template (*lower*) superimposed with the locations of significant activity in males compared with females (p<0.001, uncorrected). *Right*; glass brain and the MNI brain template superimposed with the locations of significant activities in females compared with males (p<0.001, uncorrected). MFG: middle frontal gyrus, dACC: dorsal anterior cingulate cortex, IPL: inferior parietal lobule. Numbers in parentheses are the MNI coordinates in the x, y, and z planes, respectively.

In the direct comparison between the male and female groups in the IC vs. C condition, the brain regions more activated in females than males were as follows; the left vmPFC, hippocampus, right dorsal ACC (dACC), IPL, postcentral gyrus, and lateral occipital cortex (Table 3, Fig. 4). On the other hand, the left MFG was the only region showing significantly higher activity in males than in females.

**Table 3:** Direct comparisons of gender differences in the IC vs. C condition.

Anatomical region	Males vs. Females					Females vs. Males				
	Coordinates (mm)			t	Volume (mm^3^)	Coordinates (mm)			t	Volume (mm^3^)
	x	y	z			x	y	z		
L middle frontal gyrus	−28	−4	56	4.18	240					
L ventromedial prefrontal cortex						−24	38	4	4.72	416
R anterior cingulate gyrus						24	8	36	3.85	112
						12	12	48	3.65	112
L hippocampus						−36	−20	−10	4.1	144
R postcentral gyrus						44	−20	32	3.71	128
R inferior parietal lobule						44	−42	44	3.77	96
R lateral occipital cortex						32	−76	0	4.37	336

P<0.001, uncorrected, Volume>63 mm^3^, R: right hemisphere, L: left hemisphere, MNI: Montreal Neurological Institute, x, y, z: anatomical coordinates based on the Montreal Neurological Institute brain template.

## Discussion

### Implicit and explicit self-esteem and gender-related activities in the medial prefrontal regions (vmPFC, mPFC and dmPFC)

Although we did not find any significant differences between males and females in the IAT scores and the Rosenberg scores, the averaged IAT effect was more remarkable in the female than male groups (females, *p = 0.0009*; males, *p = 0.02*). Moreover, we did not find any significant correlation between the two scores in either group, in partial agreement with a previous study showing no significant gender difference and only weak correlation between two scores [50].

In contrast, we did find remarkable gender differences in brain activity. While there were significant differences in activity between the IC and C conditions in the mPFC and dmPFC in males, such significant differences in females were observed in activities in the vmPFC. This suggests there is a gender difference in how self-referential processing is carried out in the medial prefrontal regions in the conflicts based on self-esteem. Self-reflection is the act of effortfully thinking about oneself, whereas self-knowledge is the information that is reflected upon and so retrieved [51]. While the act of self-reflection is a canonical form of controlled processing, self-knowledge consists of both automatically accessible and effortfully retrieved representations [52]–[Bibr pone.0037901-Klein2] [54]. Lieberman et al. [55] examined the neural responses of individuals who possessed strong self-schemas (i.e., automatically accessible self-knowledge) about either acting or athletics while they judged the trait descriptiveness of trait words related to acting or athletics. This study showed that retrieval of non-schematic self-knowledge was associated with activity in the dmPFC, whereas automatically accessible schematic self-knowledge was associated with activity in the vmPFC. No activity in the mPFC in this study suggests that it plays a primary role in self-reflection rather than self-knowledge [55]. Therefore, the present results showed that schematic self-knowledge was automatically retrieved in females and it was involved in vmPFC activity, while non-schematic self-knowledge was effortfully retrieved and reflected on in males and it was involved in dmPFC and mPFC activities, when responding to the incongruences. Thus, our observations in the present study suggest that females store in memory not only the congruent associations (i.e., self-positivity), but also incongruent ones (e.g., “self is negative”) as an important aspect of their schematic self-knowledge. This finding suggests that females well reflect on and memorize not only events that are comfortable or pleasurable for them, but also uncomfortable or distressful events. In fact, a somewhat related cognitive style more common in females than males is repetitive and passive ruminative thinking, focusing on symptoms of distress and their possible causes and consequences [56]. On the other hand, such incongruent associations do not appear to be as firmly fixed in the male self-knowledge as they are in females. That is, males might process the incongruent associations as being out of their schematic self-knowledge.

Northoff and Bermpohl [57] argue that the vmPFC is responsible for tagging incoming information as self-relevant, while the dmPFC functions to cognitively evaluate self-relevant information. Individuals with lower self-esteem tend to be more defensive and contribute less to their relationships with others [58], and appear to be more sensitive to rejection, sometimes perceiving rejection where it does not exist [59]. Accordingly, in the present study, the female subjects with lower explicit self-esteem may have a higher tendency to tag incongruent associations as self-relevant, leading to higher vmPFC activity. In contrast, the male subjects with higher explicit self-esteem showed higher dmPFC activity, possibly because they could process the incongruent situations more cognitively than the female subjects.

### Direct comparisons of gender differences in the IC vs. C condition

When brain activities related to the IC vs. C condition were directly compared between females and males, the vmPFC, hippocampal, dACC, postcentral, inferior parietal, and lateral occipital regions were significantly activated in females. Among these, the MNI coordinates of vmPFC (−24, 38, 4) corresponded to those that exhibited significant differences in the IC vs. C condition in the female group (Fig. 4). As discussed in the previous section, vmPFC activity is associated with automatic access to schematic self-knowledge, which is stored in a memory system that includes the hippocampus. Hippocampus is also related to emotional memory retrieval [60] [61], and its activity might be concerned with processing the episodic memories on negative emotion.

Several neuroimaging studies showed that the dACC is sensitive to goal conflicts, expectation violations, and errors in general [62] [63]. It is also sensitive to major conflicts, such as physical pain [64], salience [65], and social exclusion [66]. The dACC acts as an alarm that signals the lateral PFC to begin operating [67], and is involved in conflicts related to implicit attitudes [68]. Thus, the dACC activity we observed in females is suggestive of conflict monitoring and an alarm against the incongruent associations. In addition, this activity corresponds to the result that the averaged IAT effect, which reflects the level of conflict based on implicit self-esteem, was more remarkable in the female than male groups (females, *p = 0.0009*; males, *p = 0.02*).

Studies show that the right postcentral cortex is involved in one's own emotional feelings [69], suggesting an awareness among females of their own negative emotions in the present study. In females, the right IPL is involved in processing body shape during negative body shape self-comparison [70], and distancing when regulating emotional responses to social situations [71]. Accordingly, the right IPL activity shows the possibility that females emotionally took the incongruent associations as a result of evaluation from others on their own physical appearances which are one of the most important factors in the female self-esteem, and they control the emotional responses caused by it. By comparison, we did not observe so many significant activities in males compared to females, only in the MFG. Males might process the incongruence more cognitively than females, and the MFG activity might be therefore involved in response conflict [72].

**Figure 5:**
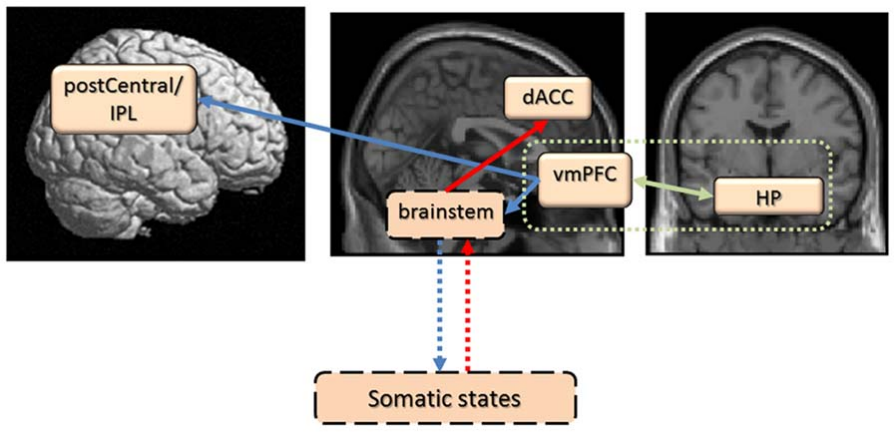
A schematic model of the female brain regions more activated than males when her self-positivity is threatened, based on the somatic-marker model which proposes the causality among brain regions involved in emotion, cognition and memory [**74**]. This shows how the brain regions, showing significantly higher activity in females than males in the present study, may have interrelationships with each other, based on the causality proposed by the somatic marker model [73]. The results on causality were not obtained in the present study. (1) Even incongruent associations (i.e., self = negative) have been firmly stored in the hippocampus and vmPFC as self-schema (surrounded with a green dot line). This information automatically acts as a secondary inducer in the face of threats (green solid line), (2) vmPFC triggers emotional (somatic) states and awareness of bodily feelings (postcentral/IPL) when the inducer is activated (blue solid lines), and (3) such somatic states influence the neural processes for emotional responses and emotion control where dACC plays an important role (red solid line). Each brain region surrounded with a solid black line is that showed significantly higher activity in females than males, in the present study, and “brainstem” and “somatic states”, each of which is surrounded with a dot black line and connected with a red dot line and a blue one, are shown based on the somatic marker model [73].

The somatic-marker hypothesis proposes that decision-making depends in many important ways on neural substrates that regulate homeostasis, emotion and feeling. Imaging studies have shown that decision-making is associated with functioning of a distributed neural network critical for the processing of emotional information, including the vmPFC, amygdala, striatum, ACC, and insular/somatosensory cortices (SI,SII), as well as non-specific neurotransmitter systems that modulate activities of neural processes involved in decision-making [73]–[74]. All the brain regions except hippocampus and lateral occipital cortex (the IPL is in or near SII) in females that were significantly activated in the direct comparison, are included the above brain regions which play main roles in the somatic-marker model. Accordingly, we would try to explain the present results of females based on the somatic-marker model which provides causal relationships among the above brain regions. Figure 5 summarizes our interpretation of the significant brain activities we observed in females, from the perspective of the somatic-marker model [73]. Here, the causal relationships among the brain regions were based on the somatic-marker model because the results on such causality were not obtained in the present study. We think that incongruent associations (i.e., self = negative) have been stored more firmly in the memory system including hippocampus as schematic self-knowledge in females than in males, through past experiences and learning. Once an external or internal cue threatening one's own self-positivity is provided, this self-knowledge acts automatically as a secondary inducer [73], based on self-esteem. The vmPFC encodes associations between secondary inducers and bioregulatory states linked to given situations in the individual experience, including bodily aspects of emotional responses, based on self-esteem. The vmPFC is also a trigger structure for emotional (somatic) states brought about by the secondary inducer, and the right postcentral/IPL region represents previous feeling and bodily states. In addition, right IPL is related to emotion regulation based on self-esteem. Such somatic states, in turn, produce some conflicts based on self-esteem, leading to heightened activity in the dACC, which is a substrate for conflict monitoring and the necessary control, and for biasing cognitions or behaviors [74].

The neural processes that were significantly activated in our female subjects may explain female cognitive/behavioral traits; females tend to ruminate more often than males, which sometimes results in a prolonged negative affect [75]–[Bibr pone.0037901-Thayer1] [77]. In addition, such traits increase the risk for depression [56]. The present study has a few limitations. As hormonal levels may have an important influence on the activity of certain brain areas [78] [79], the menstrual phases of the female subjects might have played a role in their responses, but this was not controlled. Furthermore, the activities we observed in each group are those found only in young people. It remains an issue for future studies to determine whether these associations generalize to other age groups. The neural mechanisms involved in self-esteem may be different in older and younger individuals.

Behaviors depend on the brain, and so gender differences in behaviors imply gender differences in brain structure or function. On the other hand, numerous studies report gender differences in neural activity despite no behavioral differences between males and females [80] [81]. In fact, the present study showed significantly different brain activity between males and females, although there were no significant differences in both the implicit and explicit self-esteem scores. While neural gender differences can create behavioral gender differences in some cases, neural gender differences might prevent behavioral gender differences, in other cases, by compensating for gender differences in other physiological conditions such as sex hormone levels. It is not a simple, but important and necessary task, to clarify how and why gender or sex influences brain functions. The studies on gender differences in neural activity, including the present study, may raise the possibility of early diagnosis and precise treatment and management for neurological and psychiatric disorders.
